# Designing for patient decision-making: Design challenges generated by patients with atrial fibrillation during evaluation of a decision aid prototype

**DOI:** 10.3389/fdgth.2022.1086652

**Published:** 2023-01-06

**Authors:** Janette Fanio, Erin Zeng, Brian Wang, David J. Slotwiner, Meghan Reading Turchioe

**Affiliations:** ^1^ Population Health Sciences, Weill Cornell Medical College, New York, NY, United States; ^2^ Broadmoor Solutions Inc. Sinking Spring, PA, United States; ^3^ Cerner Corporation North Kansas City, MO, United States; ^4^ Department of Cardiology, NewYork-Presbyterian Medical Group Queens, New York, NY, United States; ^5^ Columbia University School of Nursing, New York, NY, United States

**Keywords:** shared decision-making, atrial fibrillation, prototype, decision aids, iterative design, health informatics, mixed-methods

## Abstract

Shared decision-making (SDM) empowers patients and care teams to determine the best treatment plan in alignment with the patient's preferences and goals. Decision aids are proven tools to support high quality SDM. Patients with atrial fibrillation (AF), the most common cardiac arrhythmia, struggle to identify optimal rhythm and symptom management strategies and could benefit from a decision aid. In this Brief Research Report, we describe the development and preliminary evaluation of an interactive decision-making aid for patients with AF. We employed an iterative, user-centered design method to develop prototypes of the decision aid. Here, we describe multiple iterations of the decision aid, informed by the literature, expert feedback, and mixed-methods design sessions with AF patients. Results highlight unique design requirements for this population, but overall indicate that an interactive decision aid with visualizations has the potential to assist patients in making AF treatment decisions. Future work can build upon these design requirements to create and evaluate a decision aid for AF rhythm and symptom management.

## Introduction

Shared decision-making (SDM) is an increasingly embraced practice in modern medicine when there is clinical equipoise between all possible treatment options, and a patient's values and goals of care should be considered alongside the evidence about outcome (1). The SDM process is aided by the use of decision aids, which are structured tools that explicitly describe the decision to be made and present unbiased information about options, including the option of taking no action. Prior studies have well established that decision-aids improve patient knowledge, patient involvement, and decision quality (2, 3). Decision aids are commonly delivered in a digital format, which allows the information to be rapidly updated, tailored to the individual person, and more precise timing of delivery in the decision-making process (4).

Patients with atrial fibrillation (AF) could benefit from a decision aid to compare AF treatment outcomes, risks and benefits, and alignment with personal care goals. AF is the most common type of cardiac arrhythmia, and its prevalence is steadily rising (5). Treatments for AF include medications or catheter ablation, a minimally invasive procedure that involves destroying the cardiac tissue believed to be causing the arrhythmia. Both treatment pathways have their own set of associated risks, benefits, and outcomes. The decision is complicated by the fact that, while catheter ablations are recommended in evidence-based guidelines for symptomatic patients (6), patients may continue to experience persistent AF and associated symptoms even after the procedure (7, 8). Thus, the treatment choice should come from a nuanced consideration of the anticipated benefits and potential risks.

Despite being an ideal scenario for SDM, little research or decision aid development has been conducted to support patients as they choose a rhythm and symptom control strategy for AF. In fact, a recent study demonstrated that very few AF patients engage in SDM with their care teams or even understand their treatment options (9). In our previous work, we report that AF patients have unique needs that create a challenging set of design requirements—specifically, a propensity for anxiety about their cardiac status but a desire for knowledge and data (10).

With these design challenges in mind, the aim of this Brief Research Report is to describe the development and preliminary evaluation of an interactive decision aid for patients with AF. A secondary objective was to explore data visualizations for communicating the risk of outcomes from each treatment option by evaluating participants' comprehension and preferences.

## Materials and methods

### Study design

We followed the International Patient Decision Aid Standards (IPDAS) Collaboration guidelines for creating high-quality patient decision aids (3), which outlines several steps that should be taken when developing decision aids. Following the first several steps of the IPDAS guidelines, in prior work we defined the scope of the decision aid, conducted needs assessments with patients and clinicians, determined the format and distribution plan, and reviewed and synthesized evidence about treatment options as well as optimal decision aid design. We defined the scope as helping patients with AF learn about two treatment options for rhythm and symptom management, antiarrhythmic medication or catheter ablation, including how each option works and its risks and benefits. The decision aid is intended to be used by patients during a cardiac electrophysiology visit to discuss treatment options for AF, as well as before or after the visit. Our needs assessment with 15 patients and 5 clinicians underscored the need for decision aids in this specific treatment decision, and generated suggestions regarding the format and delivery of the decision aid (10). In the present study, we build on this prior work by describing the next two steps of the IPDAS guidelines: (1) prototyping and (2) alpha testing to evaluate comprehensibility and acceptability. This study was approved by the Weill Cornell Medicine Institutional Review Board.

### Prototype design and development

**Figure 1:**
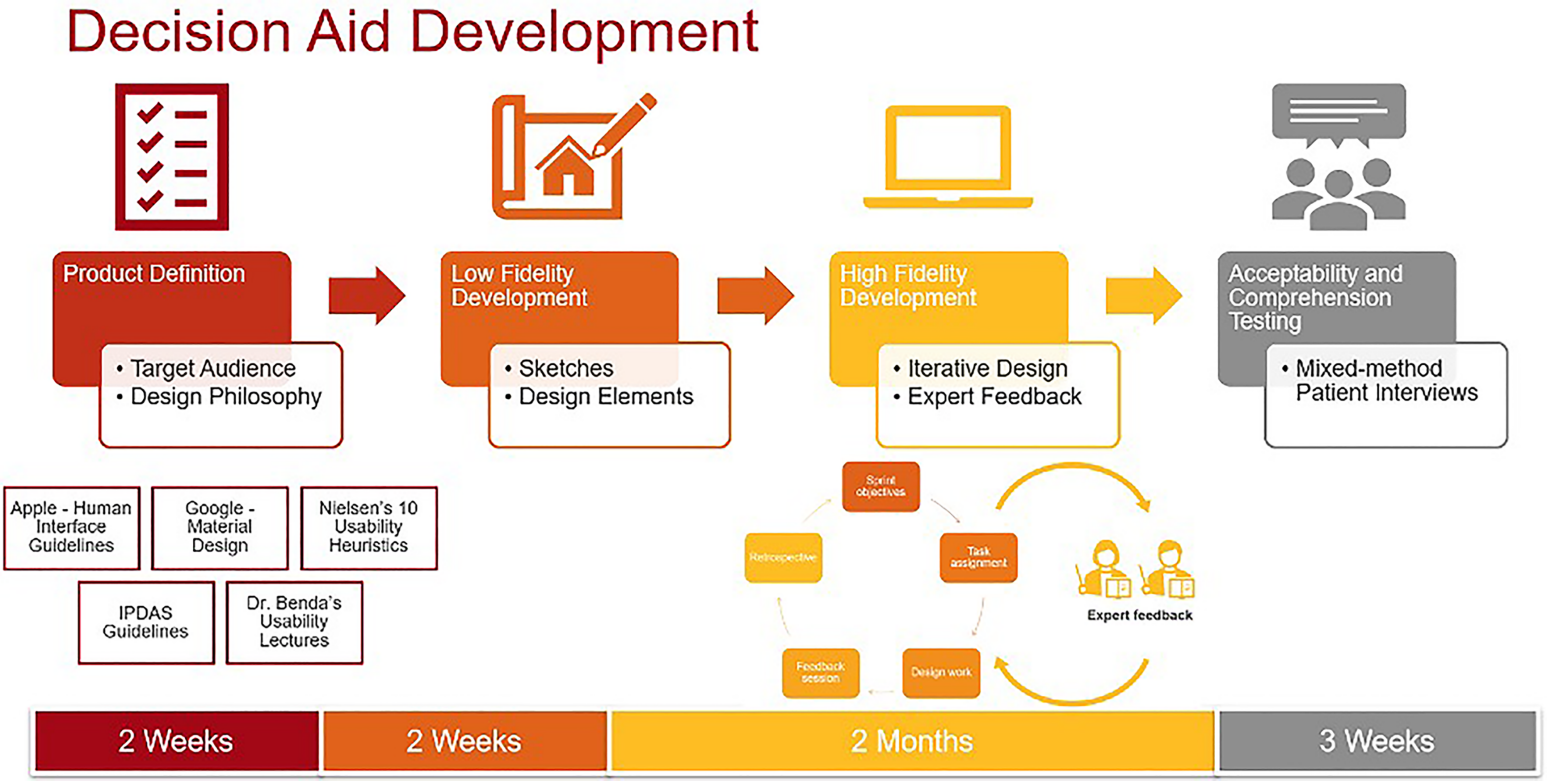
Decision Aid development.

Prototype development occurred in three phases: low-fidelity prototyping, high-fidelity prototyping, and expert feedback incorporation. Figure 1 outlines the design process. During low-fidelity prototyping we created a set of hand-drawn rough sketches, which we iterated upon until agreeing upon a design theme and common elements (Supplementary Figure S1). During this stage, we sought feedback from clinical experts who provided input on the content, color palettes, and general flow of the decision aid. We then created high-fidelity prototypes using Adobe XD, a prototyping software suite which was chosen for the purposes of creating an interactive prototype suitable for real-time collaboration and extensive version histories. We again iterated upon these prototypes until the entire research team was satisfied with the content and visual elements in the prototypes. During this stage, we sought feedback from experts in SDM, decision aid design, and data visualization, which led to further changes to the prototypes. Specifically, the experts suggested personalizing results by demographics and medical histories to avoid a “one size fits all” message to the treatment outcomes, incorporating more information about AF and treatment options so patients can explore the decision aid on their own before visits, and incorporating an open-ended question section for patients to add their preferences and questions. They also recommended studying visualizations for communicating symptoms and quality of life given the dearth of literature on this topic, as we describe below.

**Figure 2:**
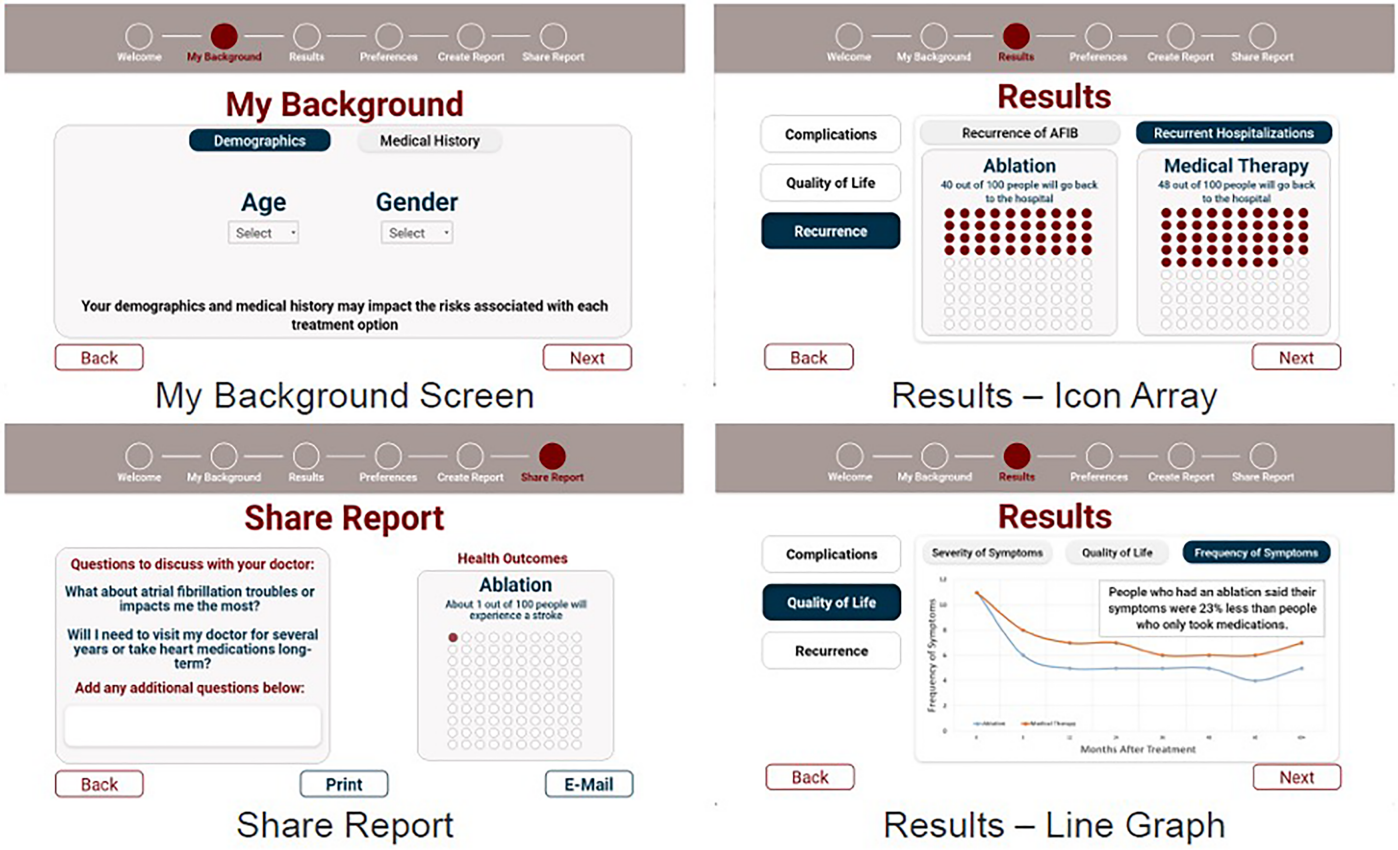
Final high-fidelity prototypes used in alpha testing.

The final interactive prototypes were used for alpha testing with patients, shown in Figure 2.

Supplementary Table S1 describes key design choices of our final prototype after incorporating feedback from our project team and external experts. In terms of user experience (UX) design, we sought out the software industry's standards for icon layout, color palette, font choices. We based our UX standards off of Apple's Human Interface Guidelines (11), Google's Material Design (12), and Nielsen's ten 10 usability heuristics (13). Accessibility and inclusive design were prioritized to ensure that the decision aid can meet the needs of a diverse target audience and includes elements such as font, size, shape, and color of each component (14). For this reason, we also followed gerontological design principles (15), such as consistent linear navigation and large touch-targets to support usability among older adults, who are the predominant age group of AF patients.

The information presented in the decision aid came from a recent meta-analysis of catheter ablation vs. medication therapy (16) and a separate clinical trial reporting symptoms and quality of life outcomes (8).

To determine how to present the information, we performed a literature review of past decision aids studies to identify evidence about which visualizations are most effective at communicating evidence. We chose to use data visualizations because numerous studies have shown that visualizations are better understood or preferred in communicating the probability of an outcome as compared to text alone (17–21). Prior studies specifically report that pictographs are the most widely comprehended visualization for communicating binary outcomes (e.g., having a stroke or not after the treatment) compared to other visualizations or text alone (17, 18, 20, 22, 23). Prior studies also recommend using the same denominator (e.g., 5 in 100 people will experience this outcome) for consistency when presenting multiple outcomes using ratios and percentages (21, 24, 25). Therefore, we adopted these visualization principles when presenting information about binary outcomes in the prototypes.

However, we found there is far less literature on how to communicate symptom experiences and quality of life in decision aids. One prior study testing the comprehension of symptom visualizations between text, text plus visual analogy (such as a gas gauge or weather icon representing symptom status), text with a number line, and text with a line graph showed that comprehension for the visual analogy was significantly higher than text alone or other visualizations (26). However, this study was focused on returning patients' personal symptom data to them, rather than projected population-level symptom outcomes in a decision aid.

Therefore, we explored comprehension of similar visualizations in the different context of SDM. Specifically, we created four visualization options showing symptom and quality of life outcomes: line graph, gauge, text with cartoon, and text alone (Supplementary Figure S2). The text alone option was the control condition. We created a version of the text alone that also included a cartoon image explaining that information should be contextualized to the individual patient, at the suggestion of experts who evaluated our high-fidelity prototypes. The gauge was selected because visual analogies were previously reported as well comprehended in older adults with cardiovascular disease (26). The line graph, although less well comprehended in prior work, most easily allowed us to display multiple data points over time. We evaluated comprehension of the four visualization options during alpha testing.

### Alpha testing

Alpha testing involves evaluating early stage prototypes with patients for usability and comprehension (27). The outcomes of interest in alpha testing were (1) objective comprehension of data visualizations included in the decision aid, measured using the International Organization for Standardization (ISO) 9,186 method (2, 28) decision aid acceptability, measured using the Decision Aid Acceptability Scale (29). We aimed to recruit 15 participants based on our prior experience with user-centered design studies and published guidance (10, 30–32), with the option to terminate recruitment early if thematic saturation in qualitative data was reached. Thematic saturation occurs when no new information is being obtained and participant responses become redundant with prior responses (33).

To conduct alpha testing, we recruited patients who had recently undergone catheter ablation at an urban hospital affiliated with New York Presbyterian-Cornell hospital in Queens, New York. The cardiology team at the hospital generated a list of potential patients, who were then contacted by phone or email and invited to participate *via* Zoom. All participants provided verbal consent to participate before each session. Each participant was compensated for their time with a $25 gift card.

During each session, we collected baseline socio-demographic information, preferences for involvement in medical decision-making measured using the Controls-Preferences Scale (34), health literacy (35), subjective numeracy (36), graph literacy (37), and experiences of decisional conflict relating to the decision to undergo ablation measured using the Decisional Conflict Scale (38).

After completing baseline surveys, participants were shown a series of screens displaying the high fidelity prototype. We collected qualitative data regarding general reactions and suggestions for improved usability, appearance, and satisfaction, and administered the Decision Aid Acceptability Scale.

Participants were then shown the four visualization options showing symptom and quality of life outcomes: line graph, gauge, text with cartoon, and text alone. The order in which visualizations were shown was randomized for each participant, known as counterbalancing, to prevent potential order effects (39). Objective comprehension was measured for each of the four visualizations.

All sessions were audio-recorded and transcribed *via* NVivo automated transcription software. The transcripts were then reviewed by two members on the research team and verified against the original recording to confirm accuracy. Qualitative data was analyzed using general thematic analysis (40). To ensure rigor in qualitative approaches, we conducted independent coding, triangulated results with quantitative surveys, and discussed results with other stakeholders to confirm credibility. To ensure rigor in qualitative approaches, we conducted independent coding, triangulated results with quantitative surveys, and discussed results with other stakeholders to confirm credibility. During the analysis, one coder analyzed the transcripts to identify themes that were reviewed and confirmed by a second coder. The emerging findings were discussed and coders independently confirmed when thematic saturation had been reached. Quantitative survey data was analyzed using basic descriptive statistics of mean, central tendency, and frequency. Qualitative and quantitative data were triangulated and the integrated findings were discussed with other key stakeholders (cardiologists and cardiac nurses) for veracity.

## Results

### Participant characteristics

Recruitment concluded after five participants were enrolled in alpha testing because thematic saturation was reached. Participants (two female and three male) had an average age of 60.2 years (SD = 7.7) (Table 1). The majority of participants were non-Hispanic/Latino White with high education levels and high technology experience. All had adequate or more than adequate financial resources and owned a laptop and an iPhone. The majority also had high willingness to engage in decision making with their care teams (controls-preferences), high health literacy, moderate subjective numeracy (mean score 13.6 out of 18, with higher scores equating to higher numeracy), but mixed levels of graph literacy.

**Table 1:** Participant characteristics (*n* = 5); mean (SD) or *n* (%).

Age	60.2 (7.7)
**Gender**	
Female	2 (40%)
Male	3 (60%)
**Race**	
White	4 (80%)
Asian	1 (20%)
Ethnicity: Not Hispanic/Latino	5 (100%)
**Education**	
High school or less	1 (20%)
College degree	2 (40%)
Master's degree	2 (40%)
**Finances**	
More than enough	2 (40%)
Enough	3 (60%)
Computer Ownership	5 (100%)
Smartphone Ownership	5 (100%)
**Internet Usage (in the last 30 days)**	
1–2 h/day	1 (20%)
5 + h/day	4 (80%)
**Controls-Preferences**	
Make the final selection after seriously considering my doctor's opinion	2 (40%)
Have my doctor and I share responsibility for deciding what treatment is best	3 (60%)
Health Literacy: adequate	5 (100%)
Subjective Numeracy	13.6 (3.0)
**Short Graph Literacy**	
1/4 Correct	1 (20%)
2/4 Correct	1 (20%)
3/4 Correct	2 (40%)
4/4 Correct	1 (20%)

### Acceptability and comprehension

The acceptability of the decision aid and objective comprehension of the visualizations are presented in Table 2. On average, the mean scores for the Welcome Page, Background and Health Results screens were higher than the Preferences, Create Report and Share Report screens, indicating higher acceptability. Four of the five participants found the decision aid to be helpful, but two thought the decision aid provided too little information to help a patient reach a treatment decision.

**Table 2:** Decision aid acceptability and visualization comprehension survey results (*n* = 5); mean (SD) or *n* (%).

Acceptability (0–5; 5 = most acceptable)	
Page 1: Welcome Page	3.2 (0.84)
Page 2: Background	3.4 (0.55)
Page 3: Health results	3.0 (1.22)
Page 4: Preferences	2.4 (1.14)
Page 5: Create Report	2.4 (1.14)
Page 6: Share Report	2.4 (1.14)
**The amount of information was:**	
Too little	2 (40%)
Just right	3 (60%)
**Would you have found this useful when you underwent an ablation?**	
Yes	4 (80%)
No	1 (20%)
**Do you think we included enough information to help a patient decide on having an ablation or not?**	
Yes	3 (60%)
No	2 (40%)
**When would you like to view this information?**	
Before you see a doctor	2 (40%)
After you see a doctor	3 (60%)
**Visualization comprehension**	
Text only	5 (100%)
Text plus cartoon	5 (100%)
Gauge	5 (100%)
Line graph	3 (60%)
**Visualization preferences**	
Text only	0
Gauge	3 (60%)
Line graph	1 (20%)
Text plus cartoon	1 (20%)

Regarding objective comprehension, all five participants correctly comprehended the text only, text plus cartoon, and gauge visualizations. Three of the five participants correctly comprehended the line graph. The majority of participants (three of the five) reported that the gauge visualization was their most preferred visualization.

### Qualitative feedback

Themes from the qualitative analysis are provided below. Illustrate quotes are provided in Supplementary Table S2.

#### Theme 1: desire for data and evidence

Most participants showed a strong desire for data and evidence, some even requesting more data than what was presented in the prototypes. All participants stated they would like to understand more about from where the evidence originated, with citations to the original trials or guidelines providing the evidence, and guidance on how they should contextualize the evidence for themselves.

#### Theme 2: preference for simplified language rather than medical terms

Since all participants were already familiar with AF and had exposure to many AF-related terms prior to the interview, they were mostly successful in comprehending the language used in the proptype. However, they still showed a preference for simplified language rather than medical terms, on some screens they required more detailed explanations about certain terms.

#### Theme 3: more details on treatment options are required

Most participants wanted more information about the treatment options available to them. One participant stated that they would like to see more treatment options other than ablation and medication, and what could be the potential outcome if the treatment did not work. Another participant suggested that patients tended to overestimate the benefits of surgical treatment and thought it would be beneficial for the decision aid to temper expectations by providing more details on potential treatment outcomes and pushing for discussions with a provider.

#### Theme 4: both digital and physical versions are important

All participants responded positively to accessing the decision-aid electronically, which participants noted was especially helpful when the COVID-19 pandemic caused anxiety around in-person visits. They also noted it facilitated communication around decision-making with care teams and caregivers. Email, text, website, participant portal and mobile app were all mentioned by participants as preferred strategies for electronically accessing a decision aid. However, participants also expressed the need to obtain physical copies of results for people with lower digital literacy, and liked having an option to print results from an electronic decision aid.

#### Theme 5: preference to use decision aid with care teams

Despite the overall high acceptability of the decision aid, participants reported a preference to review the decision aid with their doctor or other member of their care team to weigh the risks and benefits of each option. Participants were mixed regarding whether they would prefer to view the decision aid before or after consulting with their doctor.

#### Theme 6: visualizations could affect participant sentiments

Visualizations provoked both positive and negative emotional responses from participants. One participant stated that certain images in the prototype caused anxiety and triggered negative sentiments, such as the heartbeat graphic on the welcome screen. Another participant reported that viewing the cardiac outcomes caused anxiety, and that cartoon images of patients caused confusion and concern. However another participant reported that the gauge data visualization was visually appealing and lifted their mood.

## Discussion

### Summary of findings

In this study, we developed and evaluated prototypes of an AF decision aid using the steps outlined in the IPDAS guidelines for decision-aid development. Our evaluation of the interactive decision aid prototype revealed high acceptability of many pages of the decision aid. However, three important design challenges emerged: managing patient anxiety, visualizing symptom outcomes, and designing for broad accessibility. These design challenges will be critically important to address as the prevalence of AF continues to rise and the number of patients needing decision support around treatment options rises with it. In AF, many decision aids have been developed to help patients choose a stroke-preventing medication (anticoagulant); these decision aids have led to more SDM occurring between clinicians and patients and lowered patients' cognitive load and decisional conflict (41–44). Thus, well-designed decision aids for patients selecting a rhythm and symptom control strategy may have an equally positive impact on decisional outcomes. Below we describe these design challenges in greater detail and potential solutions to explore in future work.

### Challenge 1: manage patient anxiety without withholding information

Patients in our study wanted more information, but also noted how easily they could become anxious about their cardiac status. Patients requested detailed data about treatment pathways and potential adverse outcomes. At the same time, they described worrying constantly about their cardiac status and fear of those same adverse outcomes. In some cases, viewing a graphic image of a heart in our prototypes was enough to generate worry. Prior studies have indeed reported that many patients with AF struggle with anxiety symptoms (45–47). Moreover, some studies have shown that providing too much information can, in some cases, deteriorate decision quality (48).

Therefore, there exists an interesting paradox in this patient populations' information needs. Our findings suggest that patients need to see more comprehensive information presented in a straightforward manner in medical decision aids. Specifically, sources of evidence for the data being displayed should be clearly cited with hyperlinks for further reading; patients reported wanting to verify sources of data themselves. Patients also expressed a clear desire for explanations that used simple, non-medical jargon, even when they were familiar with certain medical terms. Consistent and non-medical terms are shown to reduce patient confusion (49). As in prior studies (42, 44), patients in our study strongly preferred to discuss their treatment options with their care team rather than view the decision aid independently. The context provided by healthcare professionals could also ameliorate anxiety. Finally, visualizations should be carefully examined to avoid causing anxiety and fear.

### Challenge 2: determine how to visualize symptom outcomes

Prior work has established the benefits of using visualizations to communicate evidence; patients report increased comprehension of probabilities of different outcomes occurring with each treatment option (17–23, 42). In our study, patients preferred and comprehended visualizations better than text alone. For probabilities with binary outcomes (e.g., likelihood of an adverse event occurring), studies support the use of icon arrays as the most comprehended visualization (50).

However, less is understood about the best visualizations of potential symptoms and quality of life outcomes. Symptoms and quality of life are typically measured through patient-reported outcomes measures (PROMs) which have different scoring mechanisms, making numerical comparisons difficult. For this reason, in prior studies, visual analogies such as the gauge visualization of personal PROM scores are well comprehended compared to text alone or line graphs (26). However, patients in our study reported wanting to see numerical scores, and felt that visual analogies overly simplify these measures and do not capture nuanced changes in PROMs over time. At the same time, only three of the five participants objectively comprehended line graphs (where nuanced changes were displayed in more detail), and only one participant preferred it. Adding another layer of complexity is the desire for patients to personalize data visualizations based on their personal health history, demographics, and other factors that may affect outcomes. It is possible that visual analogies paired with a “details on demand” approach, providing numerical symptom and quality of life scores plotted over time and customized to the patient, may represent a promising visualization option which should be further explored.

### Challenge 3: design for broad accessibility

Inclusive design principles ensure that applications “are accessible to, and usable by, people with the widest range of abilities within the widest range of situations” (51) and should guide every user-centered design project. While we consulted gerontological design principles (15) when creating prototypes, additional user needs and user groups should be considered. For example, the unique design needs of people with disabilities should be solicited (52). Many patients engage in SDM with the support of their caregivers (53), who should also be considered end users in usability studies.

More fundamentally, the creation of an electronic vs. a paper-based decision aid also creates barriers to access that should be carefully considered. In general, Internet use among racial and ethnic minority, low income, and older adult populations is steadily rising (54). However, one study showed that the use of digital information declined among older cohorts, but found that the physical vs. digital disparities were significantly lower among people with no college education (55). In another study, patients preferred printed medication information and had mixed responses to electronic information (56). In our study, patients preferred to have both physical and digital copies available of our decision aid's information. Creating printable screens of an electronic decision aid is one way to create broad accessibility for patients depending on their preferences.

### Strengths and limitations

In this study, we followed IPDAS guidelines closely and were able to demonstrate effectiveness and quality in the development and evaluation of the decision aid. We found success in being able to leverage several sources of widely accepted knowledge, including existing literature (for data visualization strategies), experts in atrial fibrillation and decision aids (for feedback), and industry design and heuristic standards (for our design philosophy). Our study was limited primarily by the small sample size due to thematic saturation being reached after only five participants were enrolled, which may narrow the generalizability of findings. Moreover, the sample did not include a wide range of older adults based on age or technology comfort, which may further limit generalizability. In future work we plan to refine the prototype based on the feedback provided and continue testing with larger samples of participants. This will be a critically important step to avoid creating intervention-generated inequities (57), and advance the goal of creating a highly usable and useful decision aid for AF patients to be tested in clinical trials.

## Data Availability

The raw data supporting the conclusions of this article will be made available by the authors, without undue reservation.
