# Supplementary material for: Designing for patient decision-making: Design challenges generated by patients with atrial fibrillation during evaluation of a decision aid prototype
Source: Front Digit Health. 2023 Jan 6;4:1086652. doi: 10.3389/fdgth.2022.1086652 (PMC9854261; doi:10.3389/fdgth.2022.1086652)
Supplement: Supplementary file 1 [file Datasheet1.pdf]

SUPPLEMENTARY FILE 1

Supplementary Table 1: Important High-Fidelity Prototype Design Choices And Reasons

**Design Choice**	**Description**	**Reasoning**
Data Visualization Techniques	Icon grid<br>Line graph<br>Gas gauge<br>Text only	Literature review and IPDAS guidelines showed the first three as most effective in conveying information and being comprehended.<br>Text only included as a control
Data Presentation Techniques	Point estimates of patients likely to experience each outcome	<u>Initial:</u> Point estimates for outcomes aside from quality of life; odds ratio for quality of life<br>• Best matched data presented in literature review<br><br><u>Final:</u> Adjusted all data to be presented as point estimates, including quality of life<br>• Based on expert feedback that odds ratio is confusing and potentially unethical
Preferences Questions	Determine patient's comfort level with treatments<br><br>Ask patient to identify what matters most to them<br><br>Text box for patient-entered questions	<u>Initial:</u><br>• Yes/no questions - determine if patient is OK with various treatment options or medications<br>• Based on factors from literature review<br><br><u>Final:</u><br>• All questions changed to open ended based on expert feedback<br>• Allows patient to discuss with provider
Section Ordering	Introduction<br>My Background<br>Results<br>Preferences<br>Create Report<br>Share Report	<u>Initial:</u><br>• My Background split into demographic and medical information<br>• Create and Share Report consolidated<br>• Results before Share Report<br><br><u>Final:</u><br>• Demographic and medical history questions were short enough to consolidate and save space<br>• Limited real-estate for adding more sections; needed to consolidate<br>• Preferences and report creation/sharing options were moved after Results to let patients customize what they wanted

Navigation Bar	Connected circles (representing each step) placed at the top of the screen<br><br>Current section corresponds to darker and shaded-in circle<br><br>Progresses from left to right	Designed to meet several usability heuristics<br>• *Match between system and real world* - mimics other workflow-based websites, such as tax prep software<br>• *Flexibility and efficiency of use* - instead of using next/back buttons, users can click on a section to skip<br>• *Aesthetic and minimalist design* - section names chosen to be descriptive but two words or less
Section Navigation	"Next" and "Back" buttons<br><br>Shortcuts available via the Navigation Bar	<u>Initial:</u> Created both labeled buttons and arrows on the left/right side. Arrows were intended to increase usable space<br><br><u>Final</u>: "Next" and "Back" buttons chosen to avoid confusion for less tech-savvy users
Color Palette	<u>Primary</u>: Dark Pastel Red<br><br><u>Secondary</u>: Gray, Dark Red, Blue, Light Red	<u>Initial</u>: Blue to mimic Android and Windows; Light pastel red to reflect cardiology<br><br><u>Final:</u> Muted red tone to avoid distracting patient<br>• Google Material Design's website selected secondary colors based on our primary
Font	Roboto family of fonts	<u>Initial:</u> Helvetica Neue - standard on Apple devices and commonly used<br><br><u>Final</u>: Roboto - supported on all major smartphone and computer operating systems<br>• Commonly seen on Android
Text Phrasing	Second-person language	Helps patients feel that the decision aid is patient centered and tailored for them
Vocabulary	Minimal use of abbreviations and jargon	Follows the *consistency and standards* heuristic from Nielsen to improve comprehension

Supplementary Table 2: Patient Feedback Qualitative Analysis

**Theme 1: Desire for data and evidence**	*"Yeah, a source...I think they'll probably be good if this came from like whatever overlapping studies over a certain period of time. Absolutely . I would include that. I think that's a great idea." - Patient 1*<br><br>*"...what's the evidence of this (of this) comparison? ...data points and you mean extra outside references, you mean, third party references? It's great to provide it. Absolutely. I don't know that everyone will read it, but I think it's good to have that available." - Patient 2*<br><br>*"When you say 40 out of 100. What's your timeframe for that?...But there is data, scientific data on this one. right? "- Patient 4*
**Theme 2: Preference of simplified language than medical terms**	*"When we say medical therapy, what does that mean , exactly ?...You could say medication." - Patient 1*<br><br>*"Consider your preferences needs...I think the first sentence is a little hard to read for me. Consider your own preferences and needs...maybe you just don't need that first sentence...Please select the questions that resonate with us for the first two sentences. I mean, the instructions are a little hard to read." - Patient 2*<br><br>*"...the wording is not clear. Generally speaking, would I be okay with having a minimal invasive procedure? So am I to assume that a minimally invasive procedure is the ablation?...I would have to assume that the way that's worded, it's not stated...I think the word ablation should be in there because I'm guessing if it's ablation was a minimally invasive procedure..." - Patient 3*
**Theme 3: More details on treatment outcomes are required**	*"Do you have in this study how many people take medication or have an ablation where it just doesn't work?...because I know my dad , he had it persistently for, for basically the last twenty five years of his life. He did, and he had an ablation and it didn't work. So I don't know if that's a significant enough stat. Is it 20 out of 100 or is it maybe only 1 out of 100, maybe he's a unicorn in that?" - Patient 1*<br><br>*"So it's really possible for people to, at least I did, project results that aren't based in what they've heard or what the experts are. In my case, what I was told, I was never promised that...I would be able to come off. Actually...my PCP actually,,,encouraged me to ask Dr,,,if I could come off the blood thinners...I think perhaps, perhaps maybe some more, projections, you know. Maybe to temper people's*

	optimism might be not a bad thing...I'd like to say...we're not going to make any promises here. I mean that, that might engender trust, in fact." - Patient 2<br><br>*"So I think a lot of patients have to be really made aware that this (post-ablation outcomes) is what's going to happen and everything. Because sometimes patient education is important…" Patient 4*
**Theme 4: Increasing need for obtaining both digital and physical copies**	*"...you could just send me a link, especially for this year. Once the pandemic started I think I didn't see Doctor... in person for like 15 months, 16 months. So we just did virtual appointments or something like that, just sending it over email would be great. And I'm sure some people are still, well, nervous about going into the hospital, especially in a city hospital..." - Patient 1*<br><br>*"Email it to me...but I think email would be best, you know, certainly because sometimes it's hard to get into your portal...but to repeat, I don't think it would be a bad idea to put it in the email and the portal." - Patient 2*<br><br>*"I think some people prefer the brochure, especially the older folks." - Patient 2*<br><br>*"I think an app for this could help the similar way because if you have, if you are having anxiety, you can remember Page four or page 8 because you can instantly look at your phone." - Patient 3*<br><br>*"I like having hard copies, but I also, I also enjoy doing other things like this, like this sometimes a lot of reading to do electronically. I'll print." - Patient 3*<br><br>*"...if the old patient doesn't have, even a computer or a laptop, you know, how could he do this? So maybe a print out for these people." - Patient 4*<br><br>*"I think both (a link to website and a print-out). Both." - Patient 5*
**Theme 5: Decision aid should be used in conjunction with a visit to the doctor for best results**	*"For me personally, this would be, this wouldn't do in and of itself. I would need to have conversations with professionals. But some people are different. They just like to fill out forms and see it. I'm more of a conversational type. It seems like a really good assistance tool. But I certainly wouldn't leave it out there to, to function on its own without human intervention, so to speak." - Patient 2*

	"I'm one who puts more stock in the conversation than in the...the written, than you know , than the document." - Patient 2<br><br>*"If I, if I read this before, before I met Dr...I'd get anxiety because, I'm just, my personality is...I would like to hear from the doctor first and then confirm. Like as soon as I walk out of the doctor's office I'm all over the internet." - Patient 3*<br><br>*"I think that's when your provider really has to come in and give you like, it's a double edged sword or what's the advantage and the disadvantage of having an ablation? And not having one." - Patient 4*<br><br>*you have to talk to the doctor first. Yeah. First, they don't even know what it is. So it's like all of a sudden ablation. You know, what's an ablation? - Patient 5*
**Theme 6: Visualizations could affect patient sentiments**	*"It's basically the same statement. It's just that it's, I don't know. It puts me in a better mood." - patient 2*<br><br>*"I'm opening this. I'm worrying what I'm looking, and I see this heartbeat. As opposed to something like the old fashioned heart, like a nice healthy heart... That makes me nervous...Because that makes me feel unsafe when I look at it." - Patient 3*<br><br>*"I don't necessarily get the guy with the apple. OK, if this were only men and I'm a woman now, or maybe like, maybe like a family with a mom and dad and kids. You know, I'm not really getting the guy with the apple...I think the cartoon needs to be changed... I just know something I had to picture a family was like. What are you thinking about when you're a mom or dad? What are you thinking about, about making all these decisions with your family." - Patient 3*<br><br>*"Very easy. Very, pleasant to the eye. I mean, I think the dots make it very easy to see in contrast to what's ablation versus medical therapy." - Patient 5*

Supplementary Figure 1: Low Fidelity Prototype

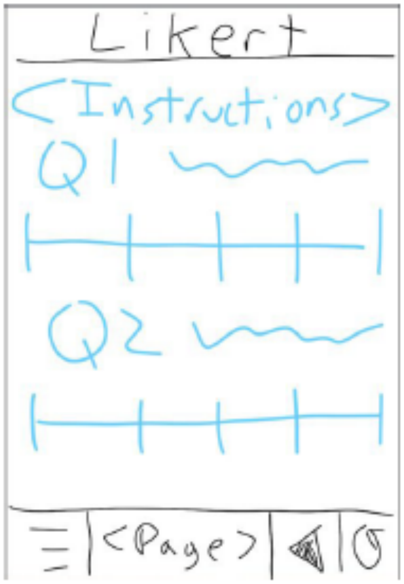

Sketch of mobile demographics page

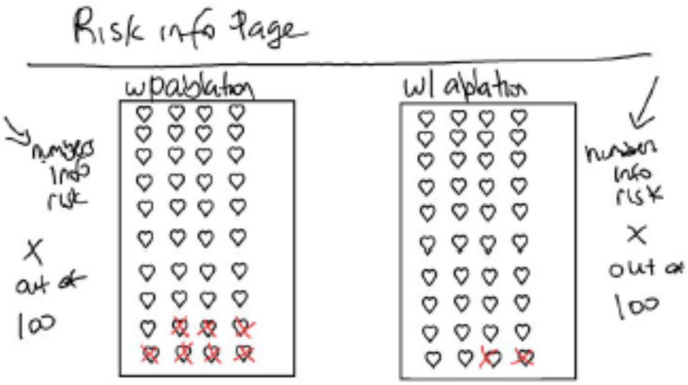

Sketch of results screen

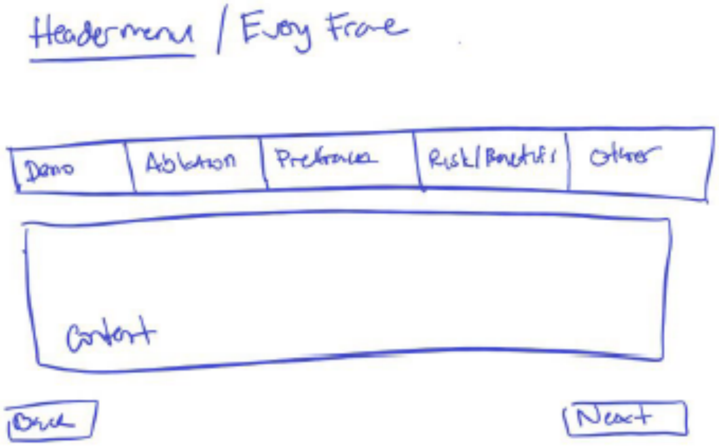

Sketch of home screen

Supplementary Figure 2: Symptom Visualizations

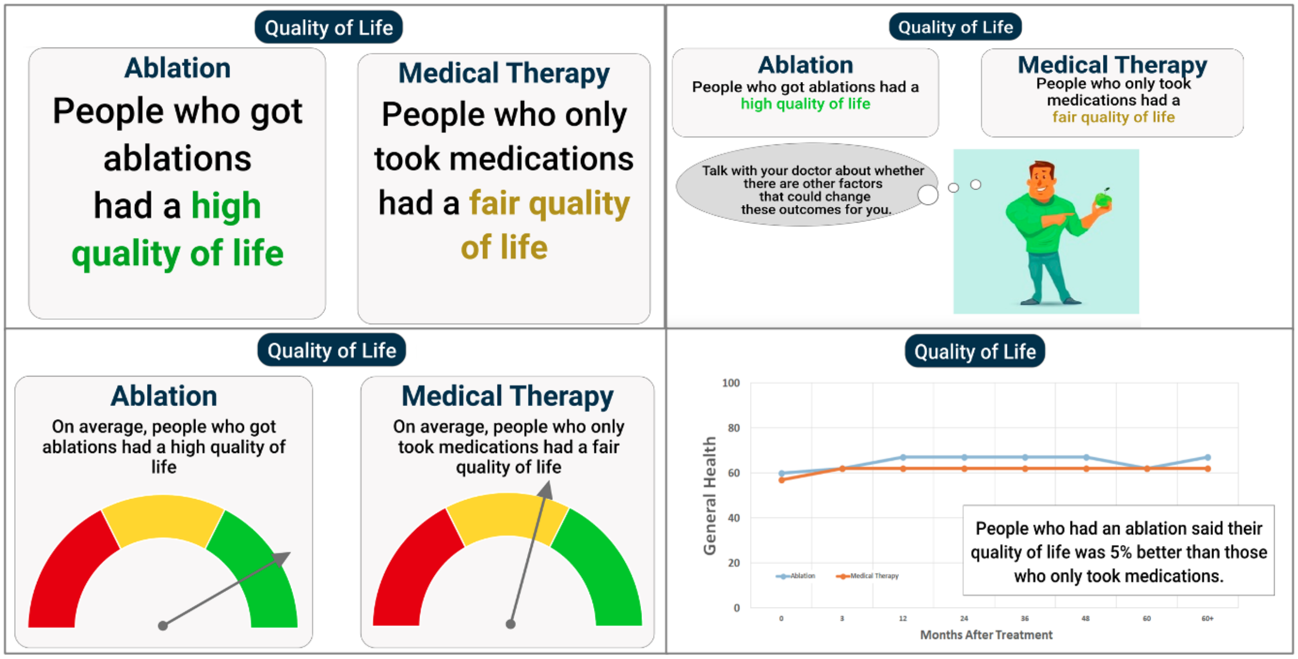

Figure legend: Symptom visualizations that were comprehension tested in alpha testing: text only (top left), text with cartoon (top right), gauge (bottom left), and line graph (bottom right).
